# Adjuvant Autologous Platelet‐Rich Plasma Gel for Decreasing Blood Loss in Posterior Placenta Previa: A Case Series and Literature Review

**DOI:** 10.1155/crog/9480876

**Published:** 2026-02-18

**Authors:** Chatuporn Duangkum, Wiyada Punjaruk, Thitinan Kasetthat, Hathairat Buraphaka, Jatupong Sitsutheechananon, Thanida Thanoorat

**Affiliations:** ^1^ Department of Obstetrics and Gynecology, Faculty of Medicine, Khon Kaen University, Khon Kaen, Thailand, kku.ac.th; ^2^ Cellular Therapy Center, Khon Kaen University, Khon Kaen, Thailand, kku.ac.th; ^3^ Department of Physiology, Faculty of Medicine, Khon Kaen University, Khon Kaen, Thailand, kku.ac.th

**Keywords:** placenta accreta spectrum, placenta previa, platelet-rich plasma, postpartum hemorrhage

## Abstract

**Abstract:**

We reported two cases of posterior placenta previa managed with elective cesarean delivery and adjunctive autologous platelet‐rich plasma (PRP) gel. Twelve milliliters of PRP gel were used to cover both the sutured implantation site and the denuded area in both cases. Case 1, gravida 1, para 0 with 37 1/7 weeks′ gestation, intra‐ and 24‐h postoperative blood loss were 400 and 10 mL, respectively, whereas the hematocrit decreased from 34.4% to 29.4%. Case 2, gravida 2, para 1, with a gestation of 37 4/7 weeks, had intra‐ and 24‐h postoperative blood loss of 500 and 50 mL, respectively. The hematocrit changed from 32.8% to 32%. No blood transfusion was required in either case.

**Conclusion:**

Adjuvant autologous PRP gel may reduce blood loss in posterior placenta previa. Further optimization of PRP preparation protocols for pregnant women, along with validation through large‐scale clinical trials, is warranted to confirm their effectiveness and generalizability.

## 1. Introduction

Placenta previa is defined as the placenta partially or completely overlying the internal cervical os, occurring in approximately 5.2 per 1000 pregnancies. [1] It increases the risk of postpartum hemorrhage, resulting from placental separation of the lower uterine segment. [2]. If placenta previa occurs in a patient with a history of cesarean delivery (CD), it is a major risk factor for placenta accreta spectrum (PAS). [3] Additional risk factors of PAS include multiparity, previous myomectomy, uterine septum excision, or curettage. [3] Concomitant placenta previa with PAS is a life‐threatening condition due to the risk of massive hemorrhage. [4, 5] Other maternal morbidity of PAS, including urological tract injury, genitourinary fistula, bowel injury, surgical site infection, as well as systemic complications such as thromboembolic events, sepsis, and acute kidney ischemia, is increased. [6] Furthermore, neonatal outcomes are markedly compromised, such as iatrogenic preterm birth, emergent delivery, low APGAR scores, respiratory distress syndrome, and higher rates of neonatal resuscitation and intensive care unit (NICU) admission. [7]

Standard treatment of placenta previa consists of scheduled CD at 36–37 weeks′ gestation, or earlier if bleeding occurs. [8] Multiple modalities, including placental bed suturing, uterine artery ligation, and uterotonic agents, are used to control bleeding from the implantation site. [1, 9, 10] However, persistent bleeding from the placental bed remains a significant challenge. It may necessitate advanced interventions such as intrauterine balloon tamponade, internal iliac artery ligation, uterine artery embolization, or cesarean hysterectomy. [9, 11]

Platelets are cytoplasmic fragments of megakaryocytes, formed in the bone marrow. [12, 13] They contain more than 30 bioactive proteins, many of which have a role in hemostasis or tissue healing. [14] After pathway activation, the platelet releases seven fundamental proteins, including platelet‐derived growth factor (PDGF), transforming growth factor‐*β*, vascular endothelial growth factor (VEGF), epidermal growth factor, and adhesive proteins such as fibrin, fibronectin, and vitronectin, to stop bleeding and promote angiogenesis. [15, 16]

Autologous platelet‐rich plasma (PRP) is derived from the patient′s own blood, which has platelet concentrations up to fivefold above baseline. [17] Given these potent effects, this study is aimed at assessing the feasibility of using autologous PRP as an adjunct to standard care for reducing intra‐ and postoperative blood loss in patients undergoing CD for posterior placenta previa.

### 1.1. Case 1

A 27‐year‐old gravida 1 para 0 with 37 1/7 weeks′ gestation underwent elective CD of total posterior placenta previa. She delivered a male infant, weighing 2790 g, with APGAR scores of 8, 9, and 9. After placental delivery, the placental bed was sutured at sites of active bleeding, followed by application of 12‐mL autologous PRP gel. The uterus was closed in two layers using 1–0 chromic catgut, and 100 *μ*g of carbetocin was administered. The operative time was 1.5 h. The estimated intraoperative blood loss was 400 mL, and a 24‐h postoperative blood loss of 10 mL; no blood transfusions were required. Preoperative hematocrit of 34.4% decreased to 29.4% at 24 h. Complete blood count (CBC) analysis showed pre‐ and postoperative platelet counts of 247 × 10^3^/*μ*L and 217 × 10^3^/*μ*L, respectively, whereas the PRP sample contained 1932 × 10^3^/*μ*L platelets and 28.6 × 10^3^/*μ*L of white blood cells.

### 1.2. Case 2

A 34‐year‐old gravida 2, para 1 underwent CD at 37 4/7 weeks′ gestation for low‐lying posterior placenta previa. A female infant, weighing 2772 g, was delivered with APGAR scores of 8, 8, and 9. Carbetocin of 100 *μ*g was administered intravenously. The placental bed was sutured at the major bleeding site, followed by application of 12‐mL autologous PRP gel. The procedure lasted for 1 h. An estimated intraoperative blood loss was 500 mL, and no blood transfusion was required. A 24‐h postoperative blood loss was 50 mL. Preoperative hematocrit and the platelet count were 32.8% and 136 × 10^3^/*μ*L, respectively. Postoperative values were 32% and 337 × 10^3^/*μ*L, respectively. The PRP sample contained 1015 × 10^3^/*μ*L platelets and 24.8 × 10^3^/*μ*L of white blood cells.

## 2. Material and Methods

The study protocol was explained to participants, and informed consent was obtained from the enrolled women. A total of 35 mL of whole blood was collected from the woman 1 h before the operation. The whole blood was divided into three portions; the first portion, 24 mL, was mixed with 6 mL of anticoagulant citrate dextrose solution, solution A (ACD‐A), the second portion, 10 mL without an anticoagulant, and the third portion, 1 mL, was evaluated as a baseline CBC using an Abbott Cell‐Dyn 3200 (Abbott Laboratories, Abbott Park, III).

**Figure 1:**
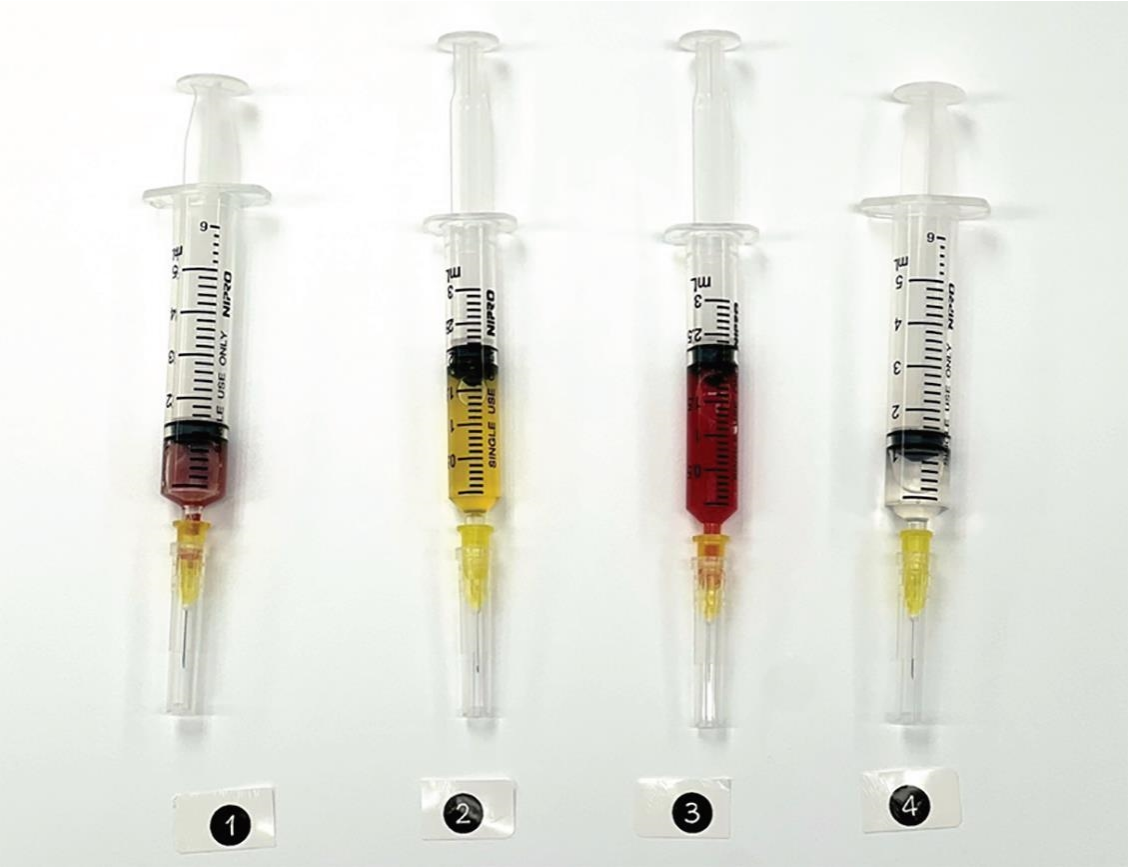
This figure illustrates four components for preparing autologous platelet rich plasma (PRP) gel. The numbered components are (1) centrifuged anticoagulated whole blood for preparing PRP, (2) dry cryoprecipitate, (3) centrifuged nonanticoagulated whole blood for preparing autologous serum, and (4) calcium gluconate.

**Figure 3:**
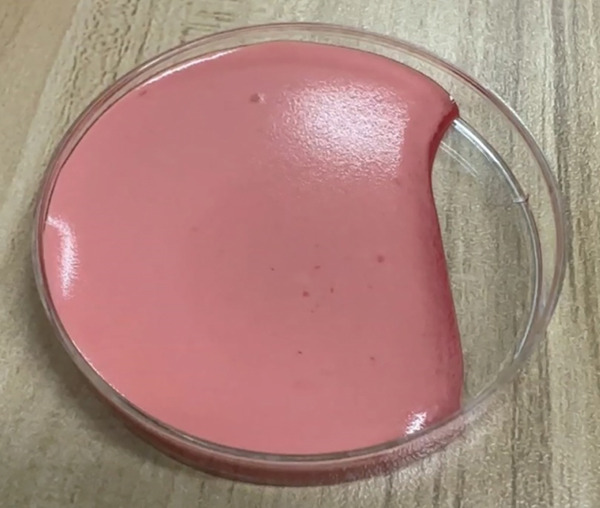
This figure demonstrates the PRP gel formation in a sterile plate after mixing for 3 minutes.

For preparing four components of PRP gel (Figure 1), PRP was harvested from anticoagulated whole blood using a double‐spin technique: the first spin involved centrifugation at 1200 RPM for 5 min, followed by a second spin at 800 RPM for 15 min. The PRP was then collected (Number 1). To enhance gel formation and strength, 2 mL of dry cryoprecipitate (Number 2) was obtained from the blood transfusion center at Srinagarind Hospital. Sample Number 3 was prepared from nonanticoagulated whole blood after centrifugation at 1000 RPM for 10 minutes, and the serum was subsequently collected. All three components were mixed, and the product was immediately activated with 1 mL of calcium gluconate (Number 4), just before clinical application, resulting in a total PRP gel volume of 6 mL. Two sets were prepared for each case. After mixing all four components, the product was immediately applied to cover the bleeding site. The autologous PRP gel formed within 3 min (Figure 2). Ex vivo gel formation also occurred within 3 min in a sterile plate after mixing (Figure 3).

Figure 2This figure illustrates the placental implantation bed (2a) and the postapplication of autologous PRP gel at the placental implantation bed (2b).

**Figure:**
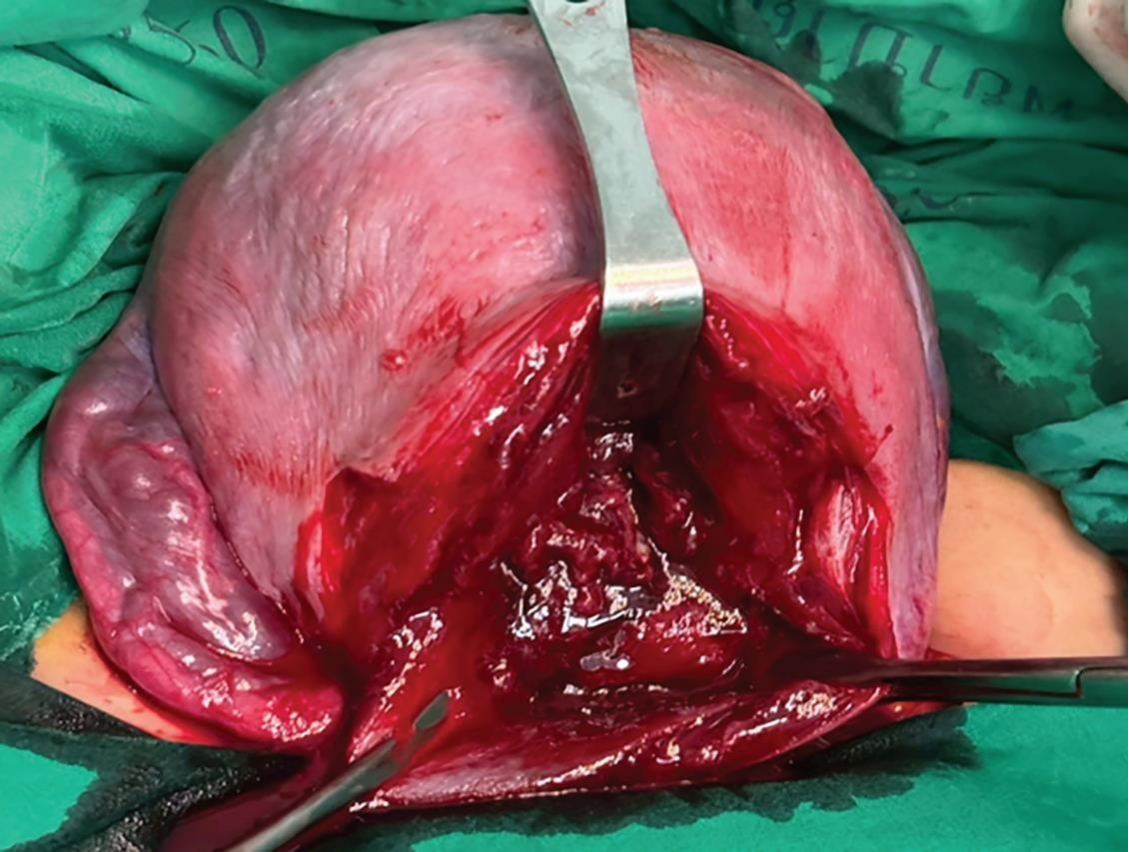
(a)

**Figure:**
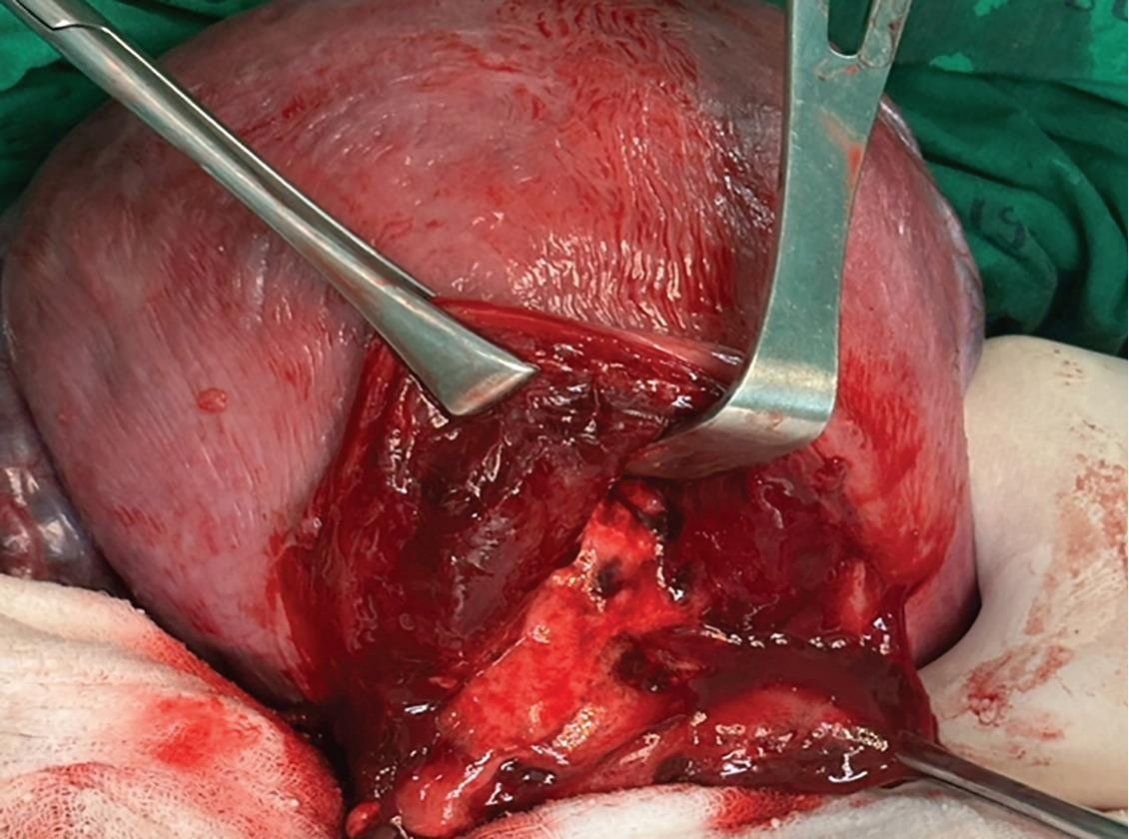
(b)

## 3. Discussion

Autologous PRP gel may function as an adjunctive therapy to reduce intra‐ and postoperative bleeding associated with placenta previa. However, the standardization of PRP preparation in pregnant women warrants optimization.

Placenta previa carries a high risk of massive hemorrhage and, in the presence of bleeding, may lead to multiple organ failure. [18] Despite multiple sutures at the placental implantation site, persistent blood loss from the placental bed still occurs due to the friable endometrium. In this study, autologous PRP gel was applied to cover both the sutured implantation site and the denuded area of the placental embedding site, providing dual therapeutic effects of hemostasis via fibrin clot formation and delivery of growth factors to promote tissue regeneration. [15, 17] After PRP activation, fibrinogen is converted to fibrin, reinforcing the platelet plug and enhancing hemostasis. [19] PDGF promotes chemotaxis of monocytes, neutrophils, and fibroblasts; stimulates smooth muscle proliferation; and supports angiogenesis, fibrous tissue formation, and reepithelialization, whereas TGF/VEGF acts as a primary inducer of angiogenesis, initiating tissue healing and regeneration. [20, 21] This study demonstrated that PRP gel may have a role in stopping bleeding, healing tissue, and enhancing angiogenesis. Postoperative blood loss was minimal, with no blood transfusion required in either case.

Autologous PRP is easily obtainable and carries no risk of disease transmission or immune rejection. However, this study had several limitations. First, surgical expertise could impact the procedure, including intraoperative bleeding and operative time. Second, the biological properties of PRP and the effects of various growth factor isoforms are not fully explained, and their roles in placental site healing require further study. Factors such as centrifugation speed, temperature, and the use of anticoagulants may influence PRP yield; therefore, an optimized protocol for pregnancy may be necessary. Additionally, more research is required to understand PRP bioformulations, platelet dosing, and the specific functions of leukocytes in hemostasis. Although this is the first study to explore PRP gel as an adjunct to standard therapy for placenta previa, further research is required to confirm these findings and evaluate their applicability, particularly in cases of placenta previa with PAS, prioritizing uterine preservation.

## 4. Conclusion

Adjuvant autologous PRP gel may reduce blood loss in posterior placenta previa. Further optimization of PRP preparation protocols for pregnant women, along with validation through large‐scale clinical trials, is warranted to confirm their effectiveness and generalizability.

## Funding

This research was funded by Khon Kaen University.

## Consent

All the patients allowed personal data processing, and informed consent was obtained from all participants.

## Conflicts of Interest

The authors declare no conflicts of interest.

## Data Availability

Data are available upon reasonable request from the corresponding author.
